# Down-Regulating γ-Gliadins in Bread Wheat Leads to Non-Specific Increases in Other Gluten Proteins and Has No Major Effect on Dough Gluten Strength

**DOI:** 10.1371/journal.pone.0024754

**Published:** 2011-09-13

**Authors:** Fernando Pistón, Javier Gil-Humanes, Marta Rodríguez-Quijano, Francisco Barro

**Affiliations:** 1 Instituto de Agricultura Sostenible, CSIC, Córdoba, Spain; 2 Universidad Politécnica de Madrid, Madrid, Spain; Tulane University, United States of America

## Abstract

**Background:**

Gliadins are a major component of gluten proteins but their role in the mixing of dough is not well understood because their contribution to wheat flour functional properties are not as clear as for the glutenin fraction.

**Methodology/Principal Findings:**

Transgenic lines of bread wheat with γ-gliadins suppressed by RNAi are reported. The effects on the gluten protein composition and on technological properties of flour were analyzed by RP-HPLC, by sodium dodecyl sulfate sedimentation (SDSS) test and by Mixograph analysis. The silencing of γ-gliadins by RNAi in wheat lines results in an increase in content of all other gluten proteins. Despite the gluten proteins compensation, *in silico* analysis of amino acid content showed no difference in the γ-gliadins silenced lines. The SDSS test and Mixograph parameters were slightly affected by the suppression of γ-gliadins.

**Conclusions/Significance:**

Therefore, it is concluded that γ-gliadins do not have an essential functional contribution to the bread-making quality of wheat dough, and their role can be replaced by other gluten proteins.

## Introduction

Wheat grain is the most important source of proteins for human beings, with 80% of the total wheat protein being represented by the gluten proteins [1]. Wheat gluten consists of over 50 different proteins, which largely determine the dough mixing properties of flours and their suitability for bread-making. These proteins are traditionally classified into the glutenins and the gliadins [2]. The glutenins comprise high molecular weight (HMW) and low molecular weight (LMW) fractions, whereas the gliadins can be divided into three structural types: α-, γ-, and ω-gliadins [1]. The glutenins are polymeric proteins stabilized by inter-chain disulfide bonds, whereas gliadins are mainly monomeric proteins that form only intra-chain disulfide bonds. The HMW subunits of glutenin (HMW-GS) have been widely studied and allelic variations in the number and composition of the HMW-GS correlated with differences in the bread-making quality of wheat [3], [4]. Although gliadins account for about 50% of the gluten proteins, their role in the mixing properties of dough is not well understood. This is because correlations between gliadin composition and the functional properties of dough are not as clear as for the HMW-GS. Gliadins are encoded by large multigene families and inherited in blocks, thus the effects of individual gliadins on dough properties are difficult to determine [5].

The functions of different protein fractions that make up the grain of cereals, especially wheat, can be understood by using mutants and by the specific silencing of individual genes or gene families using RNAi. The maize (*Zea mays*) *opaque*-*2* (*o2*) is a classic mutant of the maize kernel. The *o2* gene encodes for a basic Leucine zipper transcription factor, and mutations in this gene result in a severe reduction in the 22-kDa α-zein accumulation and in an opaque to light and floury kernel phenotype [6], [7]. The *o2* kernel also contains an elevated level of lysine [8]. When 22-kDa α-zeins were specifically suppressed in endosperm by RNA interference (RNAi), transgenic maize kernels also changed to the opaque and floury phenotype, and the lysine content was elevated in a similar manner as for the *o2* mutant, confirming that these changes are due to a reduction in 22-kDa α-zeins [9]. In the rice mutant line Low Glutelin Content-1 (LGC-1), the content of glutelin is reduced and the contents of other seed storage proteins, including prolamin, are increased [10]. Such up-regulation is not specific to the LGC-1 mutant and is thought to be a non-specific compensation for the reduction of glutelin. On the other hand, reductions of glutelins and sulfur-rich 10-kDa prolamin levels by RNAi in rice were preferentially compensated by increases of sulfur-poor and other sulfur-rich prolamins, respectively, indicating that sulfur-containing amino acids might be involved in regulating seeds storage protein composition. Furthermore, a reduction in the levels of 13-kDa, a sulfur-poor, prolamins resulted in enhancement of the total lysine content [11]. In wheat, gliadin storage proteins encoded by multigene families were down-regulated by RNAi [12], [13]. These transgenic lines are an excellent material to understand the compensatory processes that operate in the grain in response to gene silencing and to study the influence of individual groups of gliadins on the bread-making quality of wheat.

In the present study, transgenic wheat lines with the γ-gliadin fraction strongly down-regulated by RNAi were analyzed. The reversed-phase high-performance liquid chromatography (RP-HPLC), sodium dodecyl sulfate sedimentation (SDSS) test and Mixograph were used to evaluate the effect of the γ-gliadin silencing on the gluten protein composition and on technological properties of flour in the transgenic and control lines.

## Materials and Methods

### Plant material

Nine transgenic lines of the *T. aestivum* cv ‘Bobwhite 208’ (BW208) and nine transgenic lines of the *T. aestivum* cv ‘Bobwhite 2003’ (BW2003) and their corresponding wild-type lines were used in this study. Line BW2003 was found to carry the translocation T1BL.1RS. Two hairpin RNA (hpRNA) vectors were used to down regulate the γ-gliadins fraction: the pghpg8.1 vector contains the D-hordein promoter [14] while the pGghpg8.1 vector contains the γ-gliadin promoter [15]. Lines A1152, A1158, A1406, C655, C657, D445, D623, C217 and D598 contained the pghpg8.1 vector, and lines D577, D682, D715, D716, D815, 22A, 22C, 24A and 24C contained the pGhpg8.1 vector. All transgenic lines were previously reported or obtained as described by [12], [13] and self-pollinated for four generations to obtain homozygous lines.

### Reversed-phase high-performance liquid chromatography (RP-HPLC)

Gliadins and glutenins were extracted from wheat flour using a modified classical Osborne procedure based on protein solubility [16].

The gliadin fraction from 100 mg of flour was extracted stepwise three times with a 670 µl of 60% (v/v) ethanol, vortexing for 2 min at room temperature (RT) and continued with incubation at RT 10 min with shaking. Samples were centrifuged at 6,000 x *g.* for 20 min, supernatants were collected and mixed all together. Glutenin fraction was extracted from the insoluble pellet stepwise two times with 500 µl of 50% (v/v) 1-propanol, 2 M urea, 0.05 M Tris-HCl (pH 7.5) and 2% (w/v) DTT, vortexing for 2 min at RT and incubation for 15 min at 60°C with shaking. Samples were centrifuged at 6,000 x *g.* for 20 min, supernatants were collected, mixed all together and filtered through a 0.45 µm nylon filter (Teknokroma). Gliadin (40 µl) and glutenin (40 µl) extracts were applied to a 300SB-C8 reverse phase analytical column (4.6×250 mm, 5 µm particle size, 300 Å pore size; Agilent Technologies) using a 1200 Series Quaternary LC System liquid chromatograph (Agilent Technologies) with a DAD UV-V detector, as described in [13], [16]. Quantitative determination of gluten protein types in wheat flour was carried out by RP-HPLC. Absorbance was monitored with the DAD UV-V module at 210 nm. The integration procedure was handled automatically by the software with some minor manual adjustment. Absolute amounts of gliadin and glutenin fractions were determined using bovine serum albumin (BSA; BSA ≥98%, fraction V. Sigma-Aldrich, St Louis, MO, cat. no. A3294) as protein standard. Three independent repetitions were carried out for each transgenic line and control.

### “In silico” amino acid composition

Protein sequences from *T. aestivum* of each gluten protein fraction were searched in the NCBI protein database (http://www.ncbi.nlm.nih.gov/protein) using the following keywords and filter: for γ-gliadin, (γ gliadin) AND “Triticum aestivum”[porgn:__txid4565]; for ω-gliadin, (ω gliadin) AND “Triticum aestivum”[porgn:__txid4565]; for α-gliadin, (α gliadin) AND “Triticum aestivum”[porgn:__txid4565]; for HMW-GS, (HMW) AND “Triticum aestivum”[porgn:__txid4565]; and for LMW-GS, (LMW) AND “Triticum aestivum”[porgn:__txid4565]. The percentage of each amino acid was calculated for every sequence by using the bioperl script aacomp.PLS (http://www.bioperl.org/wiki/Bioperl_scripts). The average amino acid composition over the respective sequences of each gluten protein fraction, together with the amount of α-gliadins, ω-gliadins, γ-gliadins, HMW-GS and LMW-GS measured by RP-HPLC, was used to estimate the amino acid profile of each sample (Tables S1 and S2).

### Total protein analysis and sodium dodecil sulfate sedimentation test

The protein content was determined by near-infrared spectroscopy (NIRS) using a Foss-NIR Systems 6500 (NIR Systems, Inc., Maryland, USA) spectrophotometer. Protein content was expressed on a 14% moisture basis. The SDS sedimentation (SDSS) volume was determined as described by [17]. Three technical replicates were carried out for each biological sample.

### Mixograph analysis

Dough mixing properties were determined with a 10 g Mixograph (National Manufacturing Co., Lincoln NE). Prior to milling, kernel moisture was adjusted to 14% by incubation overnight at room temperature. Flour was refined through a 250 µm screen and samples were mixed to optimum water absorption following 54–40 A method [18]. The mixing parameters determined were mixing time (MT), peak resistance (PR1) or height of the centre curve at the peak point, peak width (PW1) or width of the curve at the peak point, height of the curve at three minutes after the peak (PR3), width of the curve at three minutes after the peak (PW3) and resistance breakdown (RBD) or percentage of reduction of the height between the peak point and three minutes after the peak.

### Experimental design and statistical analysis

All analyses and plot designs were conducted with the statistical software R version 2.12.1 [19] using the Graphical User Interface (GUI) R Commander. The experimental design was a randomized block design with three replications of each line, and each plot consisted of five plants. The randomized block design was generated with the package *agricolae*. Data were tested for normal distribution using Shapiro–Wilk test (function *shapiro.test*, package *stats*), and for homogeneity of variances with the Levene's test (function *leveneTest*, package *car*). Outlier data were eliminated using the function *outlierTest* (package *car*). In the cases where the conditions of data normality and homogeneity of variances were violated the Box-Cox transformation was applied (function *powerTransform*, package *car*). The differences between the control and the transgenic lines were assessed using analysis of variance (ANOVA, model “variable ∼ block + line”) with fixed effects, followed by Dunnett's *post hoc* multiple-comparison test (function *glht,* package *multcomp*).

Principal component analysis (PCA) was applied for multivariate statistical analysis. The function *fa.parallel* (package *psych*) was used to select the appropriate numbers of components for summarizing the dataset. In the following step the function *PCA* (package *FactoMineR*), with the data scaled, was used to extract the principal components.

## Results

### Gluten protein analysis

**Figure 1:**
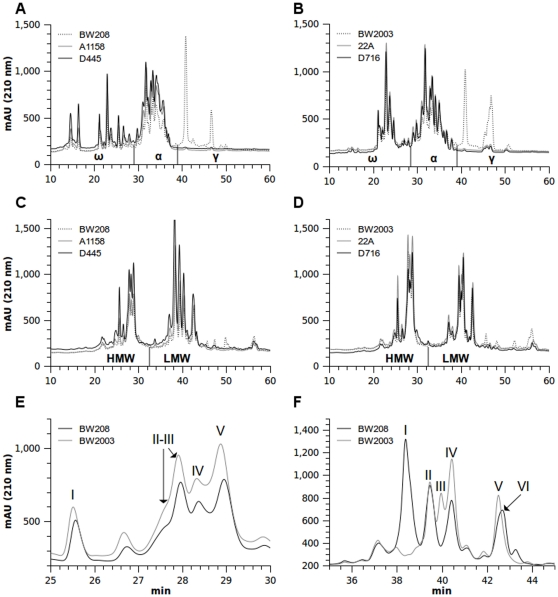
RP-HPLC chromatograms of gliadin and glutenin extracts from wild-type and transgenic wheat lines. (A) Gliadin extracts from BW208 wild-type and BW208 transgenic lines. (B) Gliadin extracts from BW2003 wild type and BW2003 transgenic lines. (C) Glutenin extracts from BW208 wild type and BW208 transgenic lines. (D) Glutenin extracts from BW2003 wild type and BW2003 transgenic lines. (E) Detail of HMW-GS fraction in the wild types BW208 and BW2003. (F) Detail of LMW-GS fraction in the wild types BW208 and BW2003. ω, ω-gliadins; α, α-gliadins; γ, γ-gliadins; HMW, high molecular weight glutenin subunit; LMW, low molecular weight glutenin subunit. Roman numbers indicate the different HMW-GS or LMW-GS peaks. mAU (210 nm), milliunits of absorbance at 210 nm; min, retention time in minutes.

In RP-HPLC proteins are eluted according to different surface hydrophobicity. The elution order is ω-, α-, and γ-type for the gliadin fraction and HMW and LMW subunits for the glutenin fraction [16], [20]. The two wild types used in this work showed clear differences in the content and pattern of the gliadin fractions. Chromatograms from transgenic lines showed a strong decrease of the peaks in the γ-gliadins region (almost null) in comparison with that of the wild types, whereas the ω and α regions increased the areas of their peaks in the transgenic lines of both genotypes (Figures 1A and 1B).

The glutenin fractions were also resolved by RP-HPLC following a similar method. We did not detect differences in the number of peaks for the HMW-GS and LMW-GS between transgenic lines and their corresponding wild types. However, there were quantitative differences as both peak heights and areas of the HMW-GS and LMW-GS regions were lower in the wild types in comparison with that of the transgenic lines (Figures 1C and D). A more detailed analysis of the HMW-GS and LMW-GS regions is shown in Figures 1E and 1F, respectively. In these chromatograms we identified the principal peaks of each region for a thorough analysis. Thus, four major peaks in the HMW region (the second one is at least the combination of two HMW-GS) and six peaks in the LMW-GS region from both genotypes were detected. As shown in Figure 1E, both BW208 and BW2003 wild-type lines shared the same HMW-GS peaks, but differed in the LMW-GS profile. However, BW208 and BW2003 shared peaks LMWII and LMWIV (Figure 1F). A small peak appeared in some RP-HPLC runs in transgenic and wild-type lines of genotypes BW208 and BW2003 at around 27.7 min retention time, which could be related to HMW-GS aggregate of two protein or a HMW-GS protein degradation product. This peak was taken into account for the total quantification of HMW-GS.

A quantitative analysis of the protein fractions was carried out by the integration of the area of each individual peak present in the chromatogram region for ω-, α-, and γ-gliadins, and the HMW-GS and LMW-GS regions from the glutenin fraction. Data were transformed to micrograms of protein per milligram of flour as showed in Table 1. All the transgenic lines showed a significant decrease in γ-gliadin content, ranging from 0.67 µg/mg of flour in line A1152 to 8.38 µg/mg of flour in line C657 (Table 1), which represented a decrease of 97.2% and 55.0%, respectively, in comparison with their respective BW208 and BW2003 wild types. The average decrease of γ-gliadin was 87% relative to the genotype BW208 and 78% relative to the genotype BW2003. The ω- and α-gliadins had a significant increase in most of the BW208 and BW2003 transgenic lines. However, the total gliadin content did not show significant differences in the transgenic lines relative to the wild types. Likewise, transgenic lines showed an increase of the HMW-GS, LMW-GS and total glutenin contents, which on average were significantly higher in BW2003 transgenic lines than in their wild type.

**Table 1:** Gliadins, glutenins, and total protein contents of transgenic and wild-type lines.

	Gliadin (µg/mg of flour)					Glutenin (µg/mg of flour)						Protein
Line	ω	α	γ	Total	α/ω	LMW	HMW	Total	L/H	S-r/S-p	Gli/Glu	(%)
BW208	14.4	36.1	24.1	74.5	2.51	15.2	9.33	24.5	1.63	5.89	3.04	10.7
A1152	18.6	46.5	0.67**	65.8	2.51	18.5	11.5	30.0	1.60	4.16**	2.19	12.0
A1158	36.6**	79.9**	1.71**	118.1	2.18	16.4	11.3	27.7	1.44	2.99**	4.13	11.4
A1406	23.9	66.2*	0.84**	90.9	2.77	13.7	12.3	26.0	1.11**	3.89**	3.45	12.6**
C655	18.4	43.2	2.77**	64.3	2.36	17.5	9.45	27.0	1.85	3.97**	2.38	11.4
C657	37.4	78.1	8.38**	123.9	2.09	17.1	9.69	26.8	1.77	3.03**	4.59	11.0
D445	59.7**	96.8**	1.22**	157.6	1.62**	20.7**	12.7	33.4	1.63	2.20**	4.98	13.2**
D577	29.3*	68.6*	4.49**	102.3	2.34	18.8	11.5	30.3	1.63	3.53**	3.42	11.6
D623	23.8	53.9	3.19**	80.9	2.27	18.0	11.2	29.2	1.6	3.63**	2.78	11.2
D682	40.1*	85.9*	5.06**	131.0	2.15	17.6	10.6	28.2	1.65	2.97**	4.65	11.0
Average	32.0**	68.8**	3.15**	103.9	2.15	17.6	11.2*	28.7*	1.58	3.15**	3.62	11.7*
Average %	222.4	190.8	13.1	139.4	85.8	115.6	119.5	117.0	96.7	53.4	119.1	109.3
BW2003	16.8	27.9	18.6	63.3	1.66	13.6	12.7	26.3	1.07	4.34	2.42	10.8
22A	42.0**	64.5**	3.23**	109.8	1.54	17.2**	19.8**	37.0**	0.87	2.49**	2.90	11.4
22C	23.9*	37.6*	1.58**	63.1	1.58	16.9*	16.1	33.0**	1.05	3.03**	1.92	11.2
24A	57.7**	84.9**	5.82**	148.4	1.47	17.8**	16.5*	34.2**	1.08	2.17**	4.60	12.5*
24B	23.7*	39.2*	1.79**	64.8	1.65	14.1	13.7	27.8	1.03	2.90**	2.35	11.8
C217	38.6**	62.8**	10.2**	111.7	1.63	16.2	17.1*	33.3**	0.95	2.75**	3.26	11.2
D598	22.0*	38.0*	6.09**	66.1	1.73	14.9	13.2	28.0	1.13	3.28**	2.35	11.0
D715	24.5*	42.3*	4.44**	71.2	1.73	16.1	15.4	31.5	1.05	3.20**	2.25	11.6
D716	24.2*	42.0**	1.98**	68.1	1.74	16.5	16.4*	32.9**	1.0	3.18**	2.07	11.8
D815	22.6*	38.5*	2.14**	63.2	1.70	16.1	15.0	31.1	1.07	3.18**	2.04	11.4
Average	31.0**	50.0**	4.14**	85.1	1.61	16.2**	15.9**	32.1**	1.02	2.78**	2.64	11.6**
Average %	184.7	178.9	22.3	134.5	96.9	119.2	125.4	122.2	95.0	64.1	109.1	107.4

Gliadins and glutenins were determined by RP-HPLC. Average, Transgenic average; Average%, Transgenic average in percent relative to control; ω, ω-gliadins; α, α-gliadins; γ, γ-gliadins; total, total gliadin content; α/ω, ratio α-gliadins/ω-gliadins; HMW, high molecular weight; LMW, low molecular weight; L/H, ratio LMW content/HMW content; S-r/S-p, ratio Sulfur-rich/sulfur-poor proteins fractions; Gli/Glu, ratio total gliadin content/total glutenin content; Protein, total protein content in percent of total flour weight.

(**) Means are significantly different to control as determined by Dunnett's multiple comparisons at P<0.05.

(*) Means are significantly different to control as determined by Dunnett's multiple comparisons at P<0.1.

The ratio α-/ω-gliadins (α/ω), gliadins/glutenins (gli/glu), and LMW-GS/HMW-GS (L/H) were not significantly affected in the transgenic lines of BW208 and BW2003, except in lines A1406 and D445 that showed significant differences relative to control for L/H and α/ω, respectively (Table 1). On the other hand, the ratio sulfur-rich/sulfur-poor fractions (S-r/S-p) was significantly different in all transgenic lines relative to wild type (Note: in sulfur-rich proteins we included α-gliadins, γ-gliadins, LMW-GS and HMW-GS, and in sulfur-poor proteins the ω-gliadins).

To detect any possible differential balancing of the individual HMW-GS and LMW-GS proteins, the main peaks of each chromatogram region were quantified individually (Table 2). The average content of the transgenic lines relative to their control showed significant increases for the peaks LMWII, LMWIV, LMWVI, HMWII-III and HMWV in the BW208 genotype; and the peaks LMWII, LMWIV, LMWV, HMWI, HMWII-III and HMWV in the BW2003 genotype. When considering lines individually, the peaks contents were similar to those showed by the average of transgenics. The LMWIV peak showed the most consistent increase in the individual transgenic lines of both genotypes.

**Table 2:** HMW and LMW glutenin subunits single peaks contents.

	LMW (µg/mg of flour)						HMW (µg/mg of flour)			
Line	I	II	III	IV	V	VI	I	II-III	IV	V
BW208	3.88	2.28	0	1.71	0	2.20	2.49	1.23	3.22	0.92
A1152	5.25**	3.27**	0	2.47**	0	2.71	2.97	1.77*	3.34	1.37*
A1158	4.64*	2.87*	0	2.24**	0	2.46	3.06	1.77*	3.39	1.36*
A1406	0.34**	2.89*	0	2.50**	0	2.97	3.23	1.85**	3.53	1.53**
C655	5.03*	3.12**	0	2.45**	0	2.60	2.60	1.35	3.11	0.98
C657	4.80*	2.95*	0	2.34**	0	2.62	2.68	1.30	3.18	1.03
D445	5.95**	3.86**	0	2.73**	0	2.97	3.44	2.00**	3.41	1.70**
D577	5.11**	3.25**	0	2.49**	0	2.76	3.18	1.64	3.67	1.25
D623	4.89*	3.09*	0	2.37**	0	2.75	3.13	1.56	3.65	1.16
D682	4.93*	3.04*	0	2.38**	0	2.65	2.83	1.38	3.37	1.13
Average	4.55	3.15**	0	2.44**	0	2.72**	3.01	1.62**	3.41	1.28**
Average %	117.4	138.2	ND	142.32	ND	123.7	120.9	131.7	105.9	139.0
BW2003	0	2.44	1.43	2.75	2.06	0	3.70	1.78	4.43	1.23
22A	0	3.38**	1.74	3.55**	2.77**	0	5.23*	2.97**	5.48	2.28**
22C	0	3.09**	1.69	3.62**	2.71**	0	4.85*	2.31	4.85	1.69*
24A	0	3.32**	1.53	3.82**	2.60**	0	4.92*	2.53**	4.93	1.83**
24B	0	2.59	1.39	3.01**	2.18	0	3.78	2.04	4.14	1.50
C217	0	3.25**	1.90**	3.33**	2.66**	0	5.08*	2.29	4.97	1.92**
D598	0	2.89*	1.57	3.09	2.31	0	3.58	1.64	3.85	1.33
D715	0	3.00**	1.57	3.52**	2.51*	0	4.56	2.32	4.91	1.61*
D716	0	3.26**	1.70	3.37**	2.63**	0	4.60**	2.30	4.60	1.68*
D815	0	2.91*	1.37	3.46**	2.44	0	4.75	2.13	4.39	1.56
Average	0	3.08**	1.61	3.42**	2.54**	0	4.60**	2.28**	4.68	1.71**
Average %	ND	126.1	112.7	124.3	122.8	ND	124.3	128.5	105.7	138.9

HMW-GS and LMW-GS peaks were determined by RP-HPLC. Roman numbers indicate individuals peaks (see Figure 1). Average, Transgenic average; Average%, Transgenic average in percent relative to control; NA, not applicable.

(**) Means are significantly different to control as determined by Dunnett's multiple comparisons at P<0.05.

(*) Means are significantly different to control as determined by Dunnett's multiple comparisons at P<0.1.

The average amino acid composition of each gluten protein fraction was calculated using the GenBank sequences (Table S1). Likewise, “*in silico*” amino acid composition of total gluten protein for each line is in Table S2. The amino acid composition did not significantly differ between transgenic and control lines.

### Dough mixing properties of the flours

The SDSS test showed no differences between the two wild-type lines, BW208 and BW2003. The average SDSS volumes of BW208 transgenic lines were not significantly different to that of the corresponding wild type. In contrast, the average SDS sedimentation volume was significantly higher in BW2003 transgenic lines than that of the wild type (Table 3).

**Table 3:** SDSS test and Mixograph parameters of flour from transgenic and wild-type lines.

	SDS	Mixograph					
Line	(ml)	MT (s)	PR1 (AU)	PW1 (AU)	PR3 (AU)	PW3 (AU)	RBD (%)
BW208	12.9	73.5	68.7	25.3	50.7	11.3	26.1
A1152	14.3	69.3	69.7	28.0**	47.0**	9.0	32.5**
A1158	12.2	57.8**	60.0**	24.0	43.3**	9.0	27.8
A1406	9.6**	53.6**	67.7	26.0	44.7**	8.3	34.0**
C655	13.8	85.1	69.0	29.0**	51.3	12.7	25.5
C657	12.6	85.1	68.0	31.5**	54.0	16.0	20.4**
D445	13.4	59.9	62.7**	29.0**	49.7	11.3	20.7**
D577	13.0	69.3	64.8**	26.0	47.7	11.0	26.4
D623	12.2	64.6	69.3	27.0**	46.8**	10.0	32.5**
D682	13.3	70.9	69.5	28.5**	51.5	10.0	25.9
Average	12.7	67.7	66.5	27.5**	48.2	10.7	27.4
Average %	98.4	92.1	96.8	108.7	95.1	94.7	105.0
BW2003	12.4	118.7	65.7	28.7	50.0	10.3	23.8
22A	14.6**	92.4**	65.0	26.7	52.0	13.7	19.9
22C	14.5**	106.1	67.0	24.3	50.7	12.7	24.3
24A	14.3**	87.2**	66.0	25	46.7	8.3	29.3
24B	12.8	79.8**	59.7	23.7	42.7**	7.0	28.4
C217	13.9	91.9**	64.3	25.7	50.7	14.3	21.3
D598	12.9	76.6**	63.0	28.7	46.7	10.3	25.3
D715	14.6**	101.6	67.5	24.5	51.0	11.5	24.4
D716	15.0**	96.6**	69.3	28.0	54.0	11.0	22.1
D815	14.6**	94.5**	65.0	29.0	49.0	10.7	24.6
Average	14.1**	91.5**	65.1	26.2	49.2	11.0	24.4
Average %	113.7	77.1	99.1	91.3	98.4	106.8	102.5

Average, Transgenic average; Average%, Transgenic average in percent relative to control. MT, mixing time; PR1, peak resistance; PW1, peak width, PR3, height at 3 min., PW3, width at 3 min.; RBD, resistance breakdown; SDSS, SDS sedimentation test.

(**) Means are significantly different to control as determined by Dunnett's multiple comparisons at P<0.05.

(*) Means are significantly different to control as determined by Dunnett's multiple comparisons at P<0.1.

**Figure 2:**
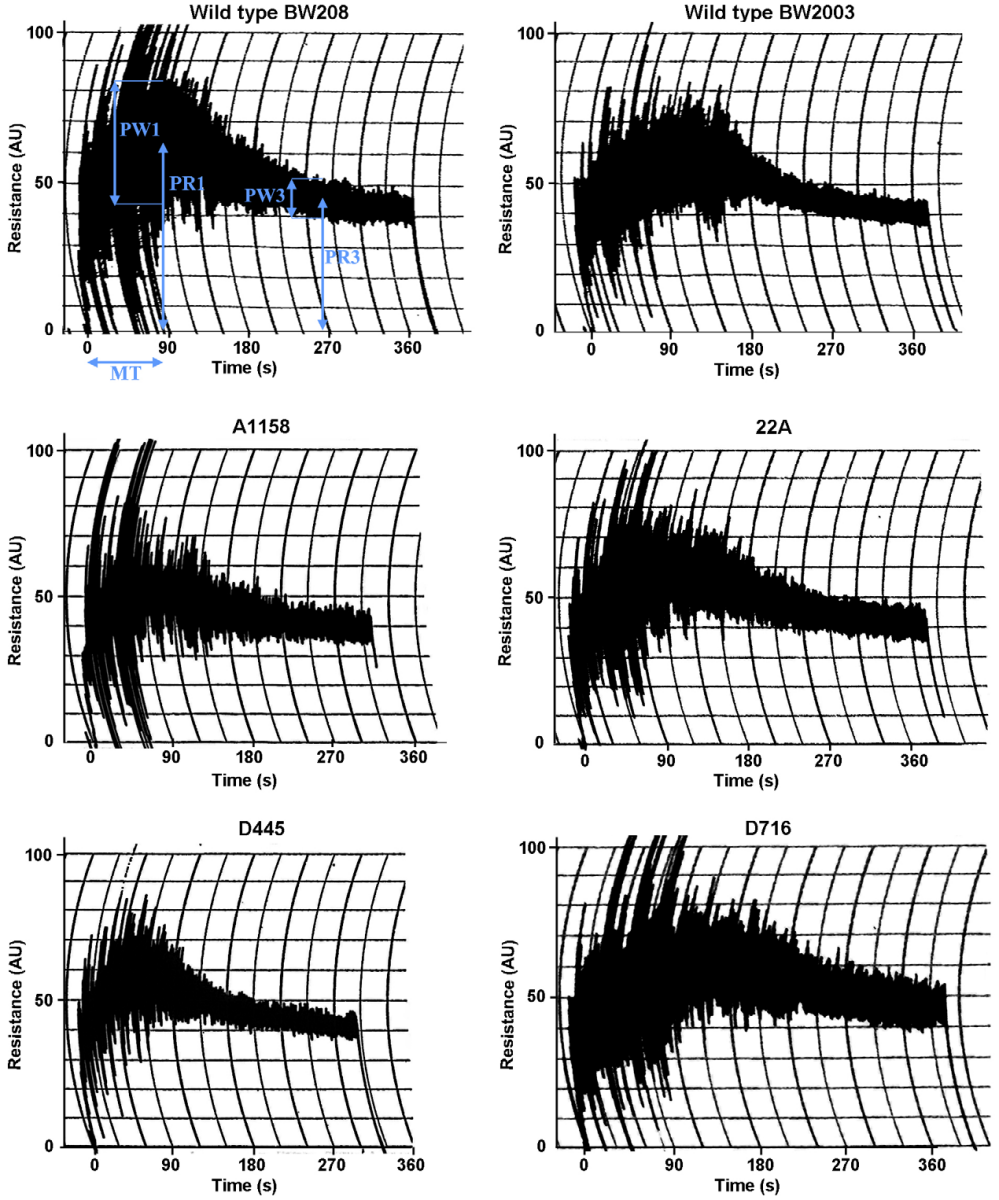
Mixograph curves of the doughs prepared from flours of the non-transformed controls BW208 and BW2003, and transgenic lines A1158, D445, 22A and D716. The parameters measured are defined in Mixograph curve from control line BW208. MT, mixing time; PR1, peak resistance; PW1, peak width; PR3, height of the curve at three minutes after the peak; PW3, width of the curve at three minutes after the peak.

The dough mixing properties of transgenic lines was analysed by using the Mixograph (Figure 2). Although there were not significant differences for Mixograph parameters between the two wild-type lines, BW208 and BW2003, their respective transgenic lines did not show the same response for these parameters (Table 3). Some individual lines showed a significant (*P<0.05)* reduction in the parameters PR1 (A1158, and D445), PR3 (A1152, A1158 and D623) and RBD (A1152, C657, D445 and D623). However, only the average value for PW1 was significantly different to that of the wild type in BW208 transgenic lines. On the other hand, the Mixograph parameter MT was significantly lower in the average BW2003 transgenic lines. Individual BW2003 transgenic lines had a similar behaviour to the wild type for all the parameters, with only the line 24B showing a significant decrease for the parameter PR3.

**Figure 3:**
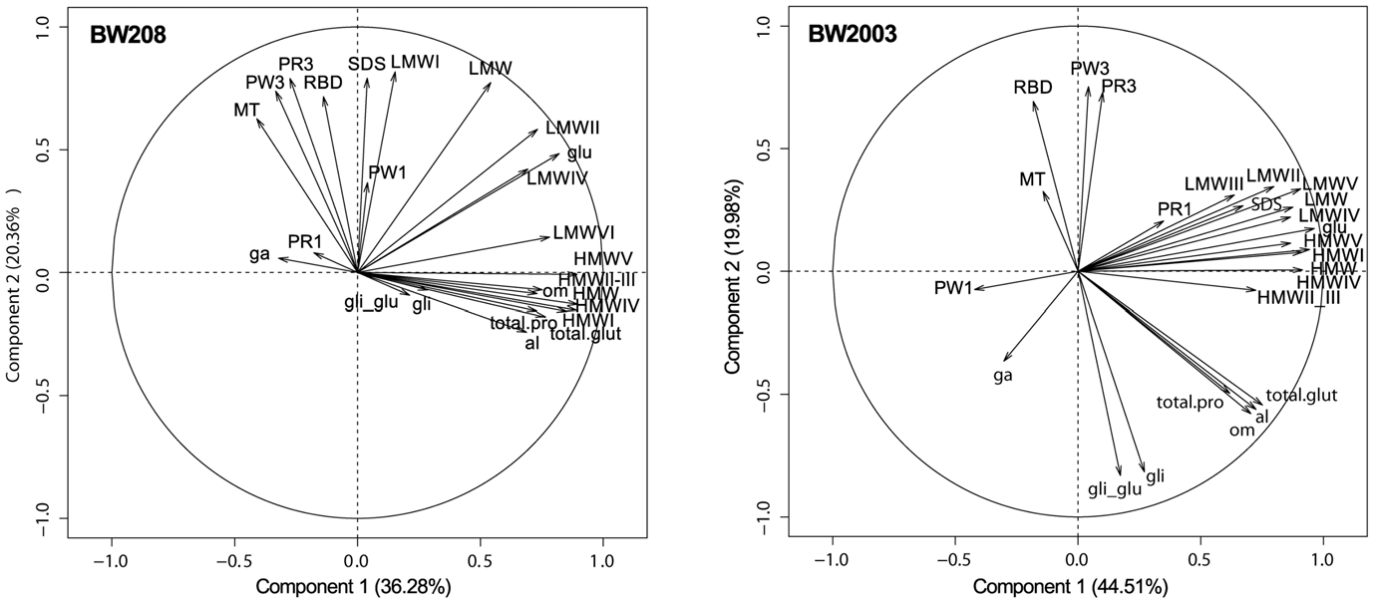
Principal component analysis (PCA) projections on axes 1 and 2 of the BW208 and BW2003 genotypes. In each figure, the eigenvalues of the correlation matrix are symbolized as vectors representing traits that most influence each axis. al, α-gliadins content; ga, γ-gliadins content; om, ω-gliadins content; gli, total gliadin content; HMW, total high molecular weight glutenin subunit content; LMW, total low molecular weight glutenin subunit content; glu, total glutenins content; total.glut, total gluten proteins content; gli_glu, ratio total gliadins content/ total glutenins content; HMW I to V, content of individual high molecular weight glutenin subunit peaks; LMW I to VI, content of individual low molecular weight glutenin subunit peaks; MT, mixing time; PR1, peak resistance; PW1, peak width, PR3, height at 3 min., PW3, width at 3 min.; RBD, resistance breakdown; SDS, SDS sedimentation test.

Principal component analysis (PCA), based on correlation matrix, was performed using the different protein fraction contents and flour quality parameters from the Mixograph tests. The analysis was carried out separately on the data from all individuals (transgenic and wild-type lines) of each BW208 and BW2003 genotype (Figure 3). In the PCA analysis of the BW208 genotype, the first two components explained 56.6% of the total variance of the dataset. The variables γ-gliadin content and PR1 were negatively correlated with the gliadin, glutenin and total protein contents. Quality parameters were mainly grouped in the second component. Thus, there was a positive correlation of MT, PW3, PR3, RBD, PW1 and SDSS test with the LMWI and total LMW-GS contents. In the BW2003 genotype, the amount of the variance explained by the first two components represented 64.5% of the total variance of the dataset. The variables γ-gliadin content and PW1 were negatively correlated with the glutenin content (total, LMW-GS and HMW-GS), the PR1 and the SDSS test. In addition, PR1 and SDSS were positively correlated. In the second component, parameters MT, RBD, PR3 and PW3 were negatively correlated with the gliadin content, the gli/glu ratio, the α- and ω-gliadins content, total protein content and total gluten protein.

## Discussion

In this study, the use of transgenic lines with γ-gliadin expression down-regulated by RNAi [12] have allowed the analysis of the influence of γ-gliadin content on flour technological properties and the examination of the interaction of γ-gliadins with other gliadin and glutenin fractions.

### Reduction of γ-gliadins is balanced by other storage proteins

The reduction of γ-gliadins in transgenic lines of both BW208 and BW2003 genotypes was accompanied by an increase in other storage proteins, in particular, the ω- and α-gliadins. The content of ω- and α-gliadins in the transgenic lines was, on average, almost twofold higher than the wild type. In addition, an increase in the average of total glutenin content was also observed, with this increase being more significant in BW2003 transgenic lines. In summary, all BW208 and BW2003 transgenic lines showed increments of ω- and á-gliadin and glutenin fractions, but the levels of increase were different depending on each transgenic line and the total gliadin content did not change significantly in transgenic lines compared with the wild types. A compensatory response to γ-gliadin down-regulation was also reflected in the total protein content, which was higher in most of transgenic lines, but more significantly for BW2003 transgenic lines.

Consistent and significant differences were found when the individual LMW-GS or HMW-GS peaks were quantified, suggesting that the compensation effect has a different intensity depending on the storage protein. In maize, reduction in zein levels (β-zein, γ-zein and their combinations) by RNAi were not compensated by increases in other types of zeins, except the suppression of 22-kDa α-zeins that was compensated by 19-kDa α-zeins, and *vice versa* [21], [22]. A sulfur-rich sunflower (*Helianthus annuus*) albumin was expressed in rice endosperm at high levels, but the total amount of sulfur-containing amino acids (Met and Cys) was not affected [23]. In these seeds, total protein content was almost constant, while each storage protein fraction changed: sulfur-rich native decreased, whereas the sulfur-poor increased. On the other hand, Kawakatsu et al. [11] reported that when a sulfur-rich RP10 was suppressed in rice endosperm, the levels of other sulfur-rich prolamins were enhanced, but the levels of sulfur-poor prolamins were depressed. They proposed that the available sulfur-containing amino acids and their key metabolic intermediates might activate sulfur-rich, or inhibit sulfur-poor, prolamin synthesis at the transcriptional or post-transcriptional level. Similar results were reported by Hansen et al. [24] in barley (*Hordeum vulgare),* who showed as the reduction of a C-hordein protein by RNAi increased the sulfur-rich B-/γ- and D-hordeins. This increment in the concentration of sulfur-rich amino acid (Cys and Met) in the transgenic lines resulted in the up-regulation of key genes in the appropriate biosynthetic pathways. Our results showed that the reduction of γ-gliadins (sulfur-rich prolamins) is compensated by other sulfur-rich prolamins (α-gliadins, HMW-GS and LMW-GS), but also by sulfur-poor prolamins (ω-gliadins). Therefore, this result does not support the hypothesis that the available sulfur-containing amino acids and their key metabolic intermediates, may activate sulfur-rich, or inhibit sulfur-poor, prolamin synthesis. Even if the sulfur-containing amino acid pathway regulation is involved in the storage protein-amino acid homeostasis in cereal endosperm, other regulatory steps are necessary to explain the stable proportion of the non-sulfur-containing amino acids and derived proteins. In the present study, the *in silico* analysis of the gluten protein amino acid composition of transgenic and control lines showed that the overall amino acids proportions are not significantly modified in transgenic lines relative to the wild types. This suggests that the developing seed maintains an appropriate amino acid content and proportion not only determined by sulfur-containing amino acids. Moreover, the ratio of sulfur-rich to sulfur-poor proteins showed significant differences between transgenic and control lines and did not maintain the proportions between them. Therefore, the observed differential compensation (higher increases in ω- and α-gliadins, and lower in HMW-GS and LMW-GS) may be determined by the availability of constituent amino acids. Storage protein compensation effects were also reported in wheat when storage proteins were over-expressed or silenced by genetic transformation [13], [25]–[27], and in aneuploid lines of hexaploid wheat (*T. aestivum*) [28]–[30], although in these earlier works the phenomenon of gluten proteins compensation was not discussed in depth.

### The reduction of γ-gliadins has little effect on dough mixing properties

The Mixograph and SDSS test are small-scale tests used to analyse dough gluten strength and mixing properties [31]-[33]. The results reported here showed significant increases in the average of SDSS volume of BW2003 transgenic lines but not in BW208 transgenic lines. Although particular LMW-GS (LMWI), gliadins and total protein content also influenced the SDSS volume, the strong reduction of γ-gliadins did not have a negative impact on the SDSS test. Furthermore, according to the SDSS values, BW2003 transgenic lines had better quality than their wild-type lines. This could be related to the higher proportions of glutenins in BW2003 transgenic lines in comparison with that of BW208. In fact, the PCA analysis for BW2003 transgenic lines showed that SDSS was mainly related to glutenin content (both total and individual proteins). The association of HMW-GS with SDSS values was reported previously [34]. The sediment in the SDS solution theoretically results from the swelling of the glutenin strands [35], and high SDSS volumes were associated with stronger gluten and superior bread-baking quality [36], [37]. Moreover, the SDSS test is a robust, highly reproducible assay and can well distinguish soft and hard hexaploid bread wheat samples based on protein quality and quantity [38]. Carter et al. [31] also reported a positive correlation between total protein content and SDSS volumes; however, the response was not consistent among all lines studied in the work.

Mixing properties of transgenic lines were analysed by using the Mixograph, which provided information on dough gluten strength closely correlated with baking quality [39]. The mixing parameters determined were mixing time (MT), peak resistance (PR1), peak width (PW1), height at three min. (PR3), width at three min. (PW3), and resistance breakdown (RBD). In general, weak gluten flour has higher RBD, and shorter MT and PR1 than strong gluten flour [40]. The MT is also negatively related with the dough extensibility [5]. The PW1 is positively correlated with extensibility [41] whereas RBD and PW2 are, respectively, negatively and positively correlated with the over-mixing tolerance (http://www.wheatflourbook.org/Main.aspx?p=36).

Most Mixograph parameters were not significantly affected by the reduction of γ-gliadins. Only the average values of PW1 in BW208 transgenic lines, and MT in BW2003 transgenic lines, showed significant differences in comparison with their wild types. Both PW1 and MT parameters are correlated with the extensibility, as described above, and therefore, the reductions of γ-gliadins increase the dough extensibility in both genotypes, probably by the increment of ω- and α-gliadins and not by the reduction in γ-gliadins. However, the reduction in the γ-gliadin content in transgenic lines does not have a major effect on the Mixograph parameters, indicating that γ-gliadins do not play a major role in dough mixing properties of wheat flour.

The dataset was analysed by PCA to examine the correlation between quality parameters and protein fractions. The Mixograph parameter PR1 is strongly correlated with HMW-GS [39]. BW208 and BW2003 have the same HMW-GS and the content is also similar, which could explain the low correlation between this parameter (PR1) and the gluten protein content in the lines studied in this work. BW208 and BW2003 showed different correlations between the rest of Mixograph parameters (MT, PR3, PW3 and RBD) and gluten proteins. In BW208 transgenic lines, the Mixograph parameters MT, PR3, PW3 and RBD were positively correlated with SDSS test and with total LMW-GS content, particularly with the LMWI peak. The association of LMW-GS with these parameters was reported previously [42]. In addition, Zhang et al. [42] showed that the *Glu*-D1 locus together with the *Glu*-B3 locus were the most important in determining the variation in Mixograph properties. The LMWI peak is only present in BW208 genotypes but not in BW2003, which contains the 1BL.1RS translocation. Hence, the LMWI peak is a strong candidate for the *Glu*-B3 locus, as this peak is correlated with the Mixograph parameters in BW208 but not in BW2003. On the other hand, in BW2003 transgenic lines, the Mixograph parameters showed a negative correlation with the gli/glu ratio and with the total gliadin content, but no association with LMW-GS or HMW-GS. Other authors have reported that the glutenin amount is significantly correlated with dough strength and bread-making quality, and the (gli/glu) ratio shows significant positive correlation with dough strength and negative correlation with dough extensibility properties [40], [43], [44]. Moreover, a more fluid gluten network resulting from a higher amount of gliadin [45] could explain the negative association between the Mixograph parameters and the ratio gli/glu and gliadin content in the lines described in this work. Finally, as for the SDSS test, there are qualitative effects on Mixograph parameters due to the LMWI peak, and quantitative effects due to the gli/glu ratio and the total gliadin content in BW208 and BW2003 lines, respectively.

### Conclusions

The strong down-regulation of γ-gliadins by RNAi in wheat lines provoked an increase in the content of other gluten proteins, specifically ω- and α-gliadins, and HMW-GS and LMW-GS. This protein compensation may be governed by the availability of amino acids, which will determine the regulation of storage proteins.

The down-regulation of γ-gliadins has no major effect on the SDSS test and on Mixograph parameters, which means a weak effect on the bread-making quality of flour. Therefore, the γ-gliadins would appear not to make an essential functional contribution to the bread-making quality of wheat dough as their role can be compensated by other gliadins or gluten proteins.

## Supporting Information

Table S1 *In silico* frequency percentage of each amino acid calculated from the sequences of the gluten proteins presents in the GenBank.
(DOC)

Table S2 *In silico* average amino acid frequency percentage of total gluten proteins by line. The average amino acid composition over the respective sequences of each gluten protein fraction, together with the amount of α-gliadins, ω-gliadins, γ-gliadins, HMW-GS and LMW-GS measured by RP-HPLC, was used to estimate the aminoacid profile of each sample. ω, ω-gliadins; α, α-gliadins; γ, γ-gliadins; total, total gliadin content; HMW, high molecular weight; LMW, low molecular weight.
(DOC)
