# Supplementary material for: Down-Regulating γ-Gliadins in Bread Wheat Leads to Non-Specific Increases in Other Gluten Proteins and Has No Major Effect on Dough Gluten Strength
Source: PLoS One. 2011 Sep 13;6(9):e24754. doi: 10.1371/journal.pone.0024754 (PMC3172295; doi:10.1371/journal.pone.0024754)
Supplement: Table S1 — In silico frequency percentage of each amino acid calculated from the sequences of the gluten proteins presents in the GenBank. (DOC) [file pone.0024754.s001.doc]

|  |  |  | Gliadin (%) | | |  | Glutenin (%) | |
| --- | --- | --- | --- | --- | --- | --- | --- | --- |
| Amino Acid | 3-Letter code |  | ω | α | γ |  | LMW | HMW |
| Alanine | Ala |  | 1.363 | 3.81 | 3.712 |  | 3.866 | 6.751 |
| Arginine | Arg |  | 1.337 | 1.869 | 1.742 |  | 2.217 | 5.151 |
| Asparagine | Asn |  | 0.603 | 2.406 | 1.991 |  | 0.819 | 0.068 |
| Aspartic acid | Asp |  | 0.207 | 0.383 | 0.96 |  | 0.237 | 1.129 |
| Cysteine | Cys |  | 0.136 | 2.057 | 2.741 |  | 2.328 | 1.966 |
| Glutamine | Gln |  | 40.182 | 32.968 | 31.189 |  | 33.257 | 20.716 |
| Glutamic acid | Glu |  | 1.951 | 1.753 | 1.043 |  | 1.733 | 4.295 |
| Glycine | Gly |  | 1.086 | 2.506 | 2.756 |  | 2.775 | 11.217 |
| Histidine | His |  | 1.061 | 2.015 | 1.665 |  | 1.373 | 0.584 |
| Isoleucine | Ile |  | 4.691 | 4.892 | 5.861 |  | 4.639 | 1.844 |
| Leucine | Leu |  | 4.208 | 8.304 | 7.224 |  | 7.951 | 7.991 |
| Lysine | Lys |  | 0.791 | 0.604 | 0.972 |  | 0.888 | 2.189 |
| Methionine | Met |  | 0.423 | 1.013 | 2.272 |  | 2.05 | 1.526 |
| Phenylalanine | Phe |  | 7.721 | 3.825 | 4.575 |  | 4.554 | 1.919 |
| Proline | Pro |  | 24.839 | 15.038 | 15.049 |  | 13.502 | 8.442 |
| Serine | Ser |  | 4.67 | 5.221 | 5.682 |  | 8.003 | 7.534 |
| Threonine | Thr |  | 2.915 | 2.729 | 3.287 |  | 3.005 | 4.636 |
| Tryptophan | Trp |  | 0.083 | 0.306 | 0.729 |  | 0.477 | 0.887 |
| Tyrosine | Tyr |  | 1.068 | 3.31 | 1.263 |  | 1.218 | 4.162 |
| Valine | Val |  | 0.666 | 4.99 | 5.232 |  | 5.109 | 6.897 |

**Table S1**. *In silico* frequency percentage of each amino acid calculated from the sequences of the gluten proteins presents in the GenBank.
