# Supplementary material for: Down-Regulating γ-Gliadins in Bread Wheat Leads to Non-Specific Increases in Other Gluten Proteins and Has No Major Effect on Dough Gluten Strength
Source: PLoS One. 2011 Sep 13;6(9):e24754. doi: 10.1371/journal.pone.0024754 (PMC3172295; doi:10.1371/journal.pone.0024754)
Supplement: Table S2 — In silico average amino acid frequency percentage of total gluten proteins by line. The average amino acid composition over the respective sequences of each gluten protein fraction, together with the amount of α-gliadins, ω-gliadins, γ-gliadins, HMW-GS and LMW-GS measured by RP-HPLC, was used to estimate the aminoacid profile of each sample. ω, ω-gliadins; α, α-gliadins; γ, γ-gliadins; total, total gliadin content; HMW, high molecular weight; LMW, low molecular weight. (DOC) [file pone.0024754.s002.doc]

|  |  | Mean (% of each aminoacid in gluten proteins) | | | | | | | | | | | | | | | | | | | |
| --- | --- | --- | --- | --- | --- | --- | --- | --- | --- | --- | --- | --- | --- | --- | --- | --- | --- | --- | --- | --- | --- |
| Line |  | Ala | Arg | Asn | Asp | Cys | Gln | Glu | Gly | His | Ile | Leu | Lys | Met | Phe | Pro | Ser | Thr | Trp | Tyr | Val |
| BW208 |  | 3.68 | 2.10 | 1.56 | 0.54 | 1.96 | 32.17 | 1.83 | 3.19 | 1.54 | 4.73 | 7.29 | 0.91 | 1.43 | 4.46 | 15.46 | 5.84 | 3.08 | 0.45 | 2.22 | 4.58 |
| A1152 |  | 3.54 | 2.13 | 1.40 | 0.40 | 1.66 | 31.53 | 2.00 | 3.19 | 1.47 | 4.25 | 7.08 | 0.85 | 1.12 | 4.31 | 15.17 | 5.68 | 2.92 | 0.35 | 2.45 | 4.22 |
| A1158 |  | 5.00 | 2.96 | 2.32 | 0.56 | 2.34 | 49.34 | 2.90 | 4.17 | 2.32 | 6.69 | 10.50 | 1.18 | 1.51 | 6.92 | 24.51 | 8.14 | 4.32 | 0.47 | 3.73 | 5.93 |
| A1406 |  | 4.24 | 2.51 | 1.87 | 0.48 | 1.98 | 38.78 | 2.40 | 3.70 | 1.86 | 5.27 | 8.63 | 0.99 | 1.26 | 5.27 | 18.90 | 6.64 | 3.51 | 0.40 | 3.13 | 5.05 |
| C655 |  | 3.31 | 1.98 | 1.35 | 0.38 | 1.58 | 30.26 | 1.85 | 2.90 | 1.41 | 4.12 | 6.71 | 0.80 | 1.08 | 4.18 | 14.63 | 5.38 | 2.77 | 0.33 | 2.27 | 3.97 |
| C657 |  | 5.11 | 2.98 | 2.42 | 0.61 | 2.48 | 51.10 | 2.90 | 4.16 | 2.40 | 7.04 | 10.80 | 1.21 | 1.64 | 7.23 | 25.43 | 8.40 | 4.46 | 0.50 | 3.70 | 6.13 |
| D445 |  | 6.20 | 3.74 | 2.89 | 0.70 | 2.84 | 65.77 | 3.78 | 5.11 | 2.96 | 8.80 | 13.30 | 1.53 | 1.88 | 9.55 | 33.42 | 10.52 | 5.63 | 0.57 | 4.64 | 7.22 |
| D577 |  | 4.68 | 2.76 | 2.08 | 0.54 | 2.24 | 44.41 | 2.64 | 3.97 | 2.09 | 6.07 | 9.67 | 1.11 | 1.48 | 6.17 | 21.77 | 7.57 | 3.97 | 0.46 | 3.35 | 5.61 |
| D623 |  | 3.95 | 2.36 | 1.66 | 0.46 | 1.87 | 36.62 | 2.23 | 3.45 | 1.70 | 4.98 | 8.03 | 0.95 | 1.26 | 5.08 | 17.87 | 6.39 | 3.33 | 0.39 | 2.76 | 4.71 |
| D682 |  | 5.40 | 3.16 | 2.56 | 0.62 | 2.58 | 54.03 | 3.10 | 4.40 | 2.54 | 7.39 | 11.43 | 1.27 | 1.68 | 7.61 | 26.89 | 8.85 | 4.70 | 0.51 | 3.99 | 6.45 |
| BW2003 |  | 3.36 | 2.02 | 1.26 | 0.50 | 1.67 | 28.90 | 1.79 | 3.19 | 1.31 | 4.11 | 6.46 | 0.88 | 1.25 | 4.08 | 14.07 | 5.34 | 2.86 | 0.41 | 2.03 | 4.05 |
| 22A |  | 5.15 | 3.22 | 2.02 | 0.63 | 2.26 | 48.97 | 3.13 | 4.86 | 2.15 | 6.48 | 10.31 | 1.34 | 1.56 | 7.02 | 24.61 | 8.38 | 4.52 | 0.51 | 3.66 | 5.91 |
| 22C |  | 3.56 | 2.25 | 1.23 | 0.43 | 1.56 | 31.45 | 2.13 | 3.52 | 1.36 | 4.13 | 6.87 | 0.93 | 1.11 | 4.43 | 15.47 | 5.73 | 3.03 | 0.37 | 2.40 | 4.09 |
| 24A |  | 6.04 | 3.70 | 2.66 | 0.73 | 2.72 | 62.31 | 3.69 | 5.25 | 2.76 | 8.33 | 12.63 | 1.54 | 1.85 | 9.09 | 31.76 | 10.12 | 5.49 | 0.58 | 4.40 | 6.97 |
| 24B |  | 3.35 | 2.10 | 1.25 | 0.40 | 1.49 | 30.56 | 2.00 | 3.21 | 1.35 | 4.05 | 6.60 | 0.87 | 1.04 | 4.32 | 15.13 | 5.42 | 2.88 | 0.34 | 2.32 | 3.87 |
| C217 |  | 5.08 | 3.11 | 2.09 | 0.65 | 2.34 | 48.35 | 2.98 | 4.64 | 2.17 | 6.55 | 10.23 | 1.30 | 1.63 | 6.92 | 24.21 | 8.25 | 4.45 | 0.53 | 3.53 | 5.93 |
| D598 |  | 3.44 | 2.12 | 1.30 | 0.43 | 1.58 | 30.92 | 1.98 | 3.25 | 1.38 | 4.18 | 6.75 | 0.88 | 1.12 | 4.36 | 15.21 | 5.54 | 2.93 | 0.37 | 2.30 | 4.03 |
| D715 |  | 3.77 | 2.34 | 1.40 | 0.47 | 1.70 | 33.70 | 2.20 | 3.62 | 1.50 | 4.51 | 7.37 | 0.97 | 1.20 | 4.74 | 16.58 | 6.05 | 3.21 | 0.40 | 2.55 | 4.39 |
| D716 |  | 3.75 | 2.35 | 1.34 | 0.45 | 1.66 | 33.04 | 2.22 | 3.67 | 1.46 | 4.37 | 7.27 | 0.97 | 1.16 | 4.63 | 16.22 | 5.99 | 3.17 | 0.39 | 2.56 | 4.33 |
| D815 |  | 3.49 | 2.19 | 1.25 | 0.42 | 1.55 | 30.89 | 2.06 | 3.40 | 1.36 | 4.09 | 6.78 | 0.90 | 1.09 | 4.33 | 15.16 | 5.60 | 2.96 | 0.36 | 2.36 | 4.04 |
|  |  |  |  |  |  |  |  |  |  |  |  |  |  |  |  |  |  |  |  |  |  |
|  |  | Standard Deviation of the above data | | | | | | | | | | | | | | | | | | | |
|  |  | Ala | Arg | Asn | Asp | Cys | Gln | Glu | Gly | His | Ile | Leu | Lys | Met | Phe | Pro | Ser | Thr | Trp | Tyr | Val |
| BW208 |  | 0.97 | 0.56 | 0.42 | 0.15 | 0.51 | 8.85 | 0.49 | 0.84 | 0.42 | 1.30 | 1.93 | 0.24 | 0.37 | 1.26 | 4.35 | 1.55 | 0.84 | 0.12 | 0.59 | 1.19 |
| A1152 |  | 0.13 | 0.08 | 0.06 | 0.01 | 0.06 | 1.43 | 0.08 | 0.11 | 0.06 | 0.19 | 0.28 | 0.03 | 0.04 | 0.21 | 0.74 | 0.23 | 0.12 | 0.01 | 0.10 | 0.15 |
| A1158 |  | 1.92 | 1.13 | 1.01 | 0.23 | 0.88 | 20.60 | 1.16 | 1.54 | 0.97 | 2.79 | 4.17 | 0.46 | 0.54 | 2.96 | 10.58 | 3.12 | 1.74 | 0.17 | 1.55 | 2.25 |
| A1406 |  | 1.02 | 0.59 | 0.53 | 0.11 | 0.47 | 10.96 | 0.61 | 0.79 | 0.52 | 1.48 | 2.23 | 0.24 | 0.29 | 1.57 | 5.58 | 1.68 | 0.91 | 0.09 | 0.81 | 1.20 |
| C655 |  | 0.45 | 0.28 | 0.19 | 0.05 | 0.20 | 4.52 | 0.28 | 0.41 | 0.20 | 0.60 | 0.94 | 0.11 | 0.14 | 0.65 | 2.27 | 0.76 | 0.40 | 0.04 | 0.33 | 0.52 |
| C657 |  | 3.41 | 1.92 | 2.02 | 0.42 | 1.67 | 40.28 | 2.02 | 2.34 | 1.91 | 5.59 | 7.83 | 0.79 | 1.04 | 5.93 | 20.93 | 5.80 | 3.24 | 0.30 | 2.74 | 4.07 |
| D445 |  | 3.30 | 1.94 | 2.02 | 0.38 | 1.50 | 43.58 | 2.18 | 2.20 | 1.96 | 5.85 | 7.90 | 0.81 | 0.91 | 6.64 | 23.28 | 5.94 | 3.37 | 0.25 | 2.84 | 3.77 |
| D577 |  | 1.35 | 0.76 | 0.79 | 0.16 | 0.65 | 15.32 | 0.80 | 0.96 | 0.74 | 2.12 | 3.05 | 0.31 | 0.39 | 2.21 | 7.90 | 2.23 | 1.24 | 0.12 | 1.11 | 1.61 |
| D623 |  | 0.43 | 0.23 | 0.26 | 0.05 | 0.21 | 4.80 | 0.24 | 0.28 | 0.24 | 0.67 | 0.98 | 0.09 | 0.12 | 0.68 | 2.45 | 0.70 | 0.38 | 0.04 | 0.36 | 0.52 |
| D682 |  | 3.02 | 1.71 | 1.79 | 0.36 | 1.46 | 35.60 | 1.80 | 2.08 | 1.69 | 4.92 | 6.93 | 0.70 | 0.90 | 5.23 | 18.49 | 5.12 | 2.85 | 0.26 | 2.46 | 3.59 |
| BW2003 |  | 0.40 | 0.22 | 0.21 | 0.07 | 0.21 | 4.07 | 0.20 | 0.32 | 0.19 | 0.60 | 0.83 | 0.10 | 0.15 | 0.60 | 2.08 | 0.65 | 0.36 | 0.05 | 0.25 | 0.49 |
| 22A |  | 2.13 | 1.30 | 1.13 | 0.27 | 0.93 | 25.07 | 1.39 | 1.72 | 1.11 | 3.34 | 4.73 | 0.55 | 0.58 | 3.77 | 13.29 | 3.62 | 2.08 | 0.18 | 1.75 | 2.41 |
| 22C |  | 0.12 | 0.08 | 0.02 | 0.01 | 0.05 | 0.57 | 0.06 | 0.15 | 0.03 | 0.07 | 0.18 | 0.03 | 0.04 | 0.07 | 0.23 | 0.16 | 0.08 | 0.01 | 0.07 | 0.14 |
| 24A |  | 3.14 | 1.82 | 1.93 | 0.38 | 1.48 | 41.41 | 2.02 | 2.06 | 1.87 | 5.64 | 7.50 | 0.78 | 0.93 | 6.34 | 22.12 | 5.67 | 3.22 | 0.26 | 2.61 | 3.63 |
| 24B |  | 0.16 | 0.12 | 0.02 | 0.02 | 0.06 | 1.35 | 0.11 | 0.20 | 0.04 | 0.16 | 0.26 | 0.05 | 0.06 | 0.22 | 0.71 | 0.28 | 0.14 | 0.02 | 0.07 | 0.17 |
| C217 |  | 2.23 | 1.31 | 1.19 | 0.30 | 1.05 | 25.02 | 1.34 | 1.76 | 1.15 | 3.45 | 4.88 | 0.56 | 0.69 | 3.71 | 13.07 | 3.72 | 2.12 | 0.22 | 1.72 | 2.61 |
| D598 |  | 0.45 | 0.25 | 0.22 | 0.06 | 0.23 | 4.10 | 0.23 | 0.37 | 0.21 | 0.59 | 0.92 | 0.10 | 0.15 | 0.56 | 2.00 | 0.69 | 0.37 | 0.05 | 0.32 | 0.55 |
| D715 |  | 0.70 | 0.44 | 0.30 | 0.08 | 0.30 | 7.23 | 0.44 | 0.63 | 0.32 | 0.94 | 1.46 | 0.18 | 0.20 | 1.05 | 3.70 | 1.17 | 0.63 | 0.06 | 0.53 | 0.80 |
| D716 |  | 0.29 | 0.18 | 0.11 | 0.04 | 0.13 | 2.31 | 0.16 | 0.29 | 0.11 | 0.31 | 0.54 | 0.07 | 0.09 | 0.31 | 1.10 | 0.43 | 0.23 | 0.03 | 0.20 | 0.34 |
| D815 |  | 0.10 | 0.06 | 0.02 | 0.01 | 0.05 | 0.41 | 0.04 | 0.12 | 0.02 | 0.06 | 0.15 | 0.02 | 0.04 | 0.06 | 0.21 | 0.12 | 0.05 | 0.01 | 0.06 | 0.13 |

**Table S2**. *In silico* average amino acid frequency percentage of total gluten proteins by line. The average amino acid composition over the respective sequences of each gluten protein fraction, together with the amount of α-gliadins, ω-gliadins, γ-gliadins, HMW-GS and LMW-GS measured by RP-HPLC, was used to estimate the aminoacid profile of each sample. ω, ω-gliadins; α, α-gliadins; γ, γ-gliadins; total, total gliadin content; HMW, high molecular weight; LMW, low molecular weight.
